# Multivariate Neuromuscular Profile and Principal Component Analysis: A Pilot Study in Professional Female Soccer Players

**DOI:** 10.3390/life16081248

**Published:** 2026-07-28

**Authors:** Luis Romero-Vera, David Ulloa-Díaz, Jonathan Concha-Poblete, Misael Sandoval-Cárcamo, Claudio Carvajal-Parodi, Francisco Guede-Rojas, Carlos Jorquera-Aguilera, Cristobal Ulloa-Barboza, Claudio Hernández-Mosqueira, Gustavo Pavez-Adasme, Eduardo Guzmán-Muñoz, Héctor Fuentes-Barría, Jorge Leschot-Gatica

**Affiliations:** 1 Department of Sports Sciences and Physical Conditioning, Universidad Católica de la Santísima Concepción, Concepción 4030000, Chile; luis.romero@ucsc.cl; 2 Carrera de Pedagogía en Educación Física, Facultad de Educación, Universidad Católica de la Santísima Concepción, Concepción 4030000, Chile; jconcha@educfisica.ucsc.cl; 3 Facultad de Educación, Universidad de Concepción, Concepción 4030000, Chile; misael.sandovalc@gmail.com; 4 Escuela de Kinesiología, Facultad de Ciencias de la Rehabilitación y Calidad de Vida, Universidad San Sebastián, Concepción 4030000, Chile; claudio.carvajal@uss.cl; 5 Exercise and Rehabilitation Science Institute, School of Physical Therapy, Faculty of Rehabilitation Science, Universidad Andres Bello, Santiago 7591538, Chile; francisco.guede@unab.cl; 6 Facultad de Ciencias, Escuela de Nutrición y Dietética, Universidad Mayor, Santiago 8580745, Chile; 7 Carrera de Ingeniería Civil Informática, Facultad de Ingeniería, Universidad Católica de la Santísima Concepción, Concepción 4030000, Chile; culloab@ing.ucsc.cl; 8 Departamento de Ciencias de la Educación, Universidad del Bío-Bío, Chillán 3780000, Chile; chernandez@ubiobio.cl; 9 Regional Center for Advanced Studies in Active and Healthy Lifestyles, Universidad Adventista de Chile, Chillán 37800000, Chile; gustavopavez@unach.cl; 10 Escuela de Pedagogía en Educación Física, Facultad de Educación, Universidad Autónoma de Chile, Talca 3460000, Chile; eduardo.guzman@cloud.uautonoma.cl; 11 Centro de Investigación en Medicina de Altura (CEIMA), Universidad Arturo Prat, Iquique 1110939, Chile; hefuentes@unap.cl; 12 Facultad de Salud y Ciencias Sociales, Escuela de Ciencias de la Actividad Física, Universidad de Las Américas, Concepción 4030000, Chile; jleschotg@gmail.com

**Keywords:** female soccer, principal component analysis, neuromuscular profile, force platform, lower-limb muscle imbalance

## Abstract

Background: Professional women’s football requires precise neuromuscular characterisation because of the high mechanical demands associated with acceleration, deceleration, jumping, braking, landing, and rapid force production. The aim was to characterise the multivariate neuromuscular profile derived from the countermovement jump (CMJ) and isometric mid-thigh pull (IMTP), and to examine neuromuscular components according to tactical positions in professional female football players. Methods: Twenty-three professional female football players were assessed using the CMJ and IMTP. Force-time, impulse, rate of force development, jump performance, landing, and inter-limb imbalance variables were extracted. Principal component analysis (PCA) was applied to the standardised variables, and individual component scores were compared according to tactical positions using one-way analysis of variance (ANOVA). Results: The first five principal components explained 79.19% of the total variance. PC1 represented the propulsive force and the rate of force development; PC2 reflected total force and mechanical impulse; PC3 represented early explosiveness; PC4 grouped indicators of inter-limb imbalance; and PC5 integrated jump performance and landing control. No statistically significant differences were observed between tactical positions. Conclusions: The combination of CMJ, IMTP, and PCA enabled multiple muscle-force variables to be synthesised into interpretable neuromuscular dimensions. The findings provide preliminary and exploratory evidence to support the individual monitoring of female football players. Given the low participant-to-variable ratio, the PCA findings should be considered exploratory.

## 1. Introduction

Professional female soccer has undergone sustained competitive, physical, and methodological development, increasing the need to characterise players’ neuromuscular profiles with greater precision [1,2]. Match demands include repeated accelerations and decelerations, changes in direction, jumps, braking actions, physical contacts, and rapid force production within short time windows, such as 100, 150, and 200 ms [3,4]. These actions depend on maximal isometric strength, propulsive force, rate of force development, eccentric control, intersegmental coordination, and landing stability [5,6]. The isolated assessment of a single variable limits the understanding of neuromuscular performance, given the multifactorial nature of soccer-specific actions [7,8]. Therefore, the applied characterisation of the neuromuscular profile requires the integration of mechanical, temporal, and bilateral variables within a common interpretative framework [9,10].

Lower-limb strength and power are relevant mechanical determinants of physical performance in soccer players [11,12]. High force production during the propulsive phase of the CMJ contributes to vertical jumping, initial acceleration, changes of pace, and high-intensity locomotor actions [13,14]. The rate of force development allows the capacity of the neuromuscular system to generate force rapidly to be quantified, which is a critical quality in actions with limited time for mechanical application [15,16]. Force-time variables, such as peak force, mean force, impulse, and force recorded at specific time intervals, allow differentiated mechanical strategies during explosive tasks to be described [17,18]. These metrics provide complementary information on force magnitude, speed of force application, movement efficiency, and the temporal organisation of mechanical production [19,20].

Bilateral force platforms allow the CMJ to be assessed in greater depth than other jump devices because they record the force-time curve and enable specific phases of the movement to be segmented [21,22]. From the CMJ, indicators of the unweighting phase, braking phase, propulsive phase, flight phase, landing phase, and post-contact stabilisation can be obtained [23,24]. These indicators include, among others, braking force, propulsive force, impulse, flight time, jump height, take-off velocity, landing stiffness, and time to stabilisation [25,26]. The availability of multiple variables increases the diagnostic capacity of the assessment, but also increases analytical complexity, redundancy between correlated indicators, and the difficulty of applied interpretation [27,28].

The isometric mid-thigh pull (IMTP) is a widely used test for assessing maximal isometric strength and rate of force development under standardised postural conditions [29,30]. Unlike jump-derived metrics, the IMTP allows force production capacity to be analysed without the direct influence of flight mechanics, landing strategy, or CMJ-specific coordination [31,32]. In soccer players, this test provides relevant information on the ability to generate high levels of force during early contraction intervals, which may be related to accelerations, braking actions, changes in direction, and high-intensity neuromuscular actions [33,34]. Therefore, the combination of CMJ and IMTP enables complementary dimensions of neuromuscular performance to be characterised: dynamic force production, propulsive capacity, maximal isometric strength, and early explosiveness [35,36].

Principal component analysis (PCA) represents a useful multivariate strategy for reducing the dimensionality of large sets of biomechanical variables [37,38]. This technique transforms correlated variables into a smaller number of latent components, retaining the greatest possible proportion of the original variance [39,40]. In sports biomechanics, this approach allows underlying neuromuscular dimensions to be identified from force, impulse, rate of force development, jump, inter-limb imbalance, and landing variables [41,42]. Principal components can synthesise mechanical profiles associated with propulsive capacity, force magnitude, early explosiveness, bilateral force distribution, and post-landing control [43,44]. This approach reduces reliance on isolated univariate indicators and favours a more integrated interpretation of neuromuscular performance in sports monitoring contexts [45,46].

Compared with traditional correlation or regression analyses, which evaluate specific associations between selected variables and generally require the prior definition of predictor and outcome variables, PCA simultaneously examines the covariance structure of a broad set of correlated indicators [37,38,39,40,41,42,43,44,45,46]. Organising variables into interpretable components may provide useful information for longitudinal monitoring. Changes from an individual athlete’s profile could help contextualise neuromuscular fatigue, guide the selection of training content, and complement monitoring during injury-prevention and return-to-play processes. However, the components should not be considered diagnostic markers or independent predictors of injury; their interpretation requires integration with clinical history, training load, competitive exposure, and functional progression [9,45].

The available evidence in professional female soccer players remains limited regarding the integrated use of CMJ, IMTP, and force platforms to characterise neuromuscular profiles, particularly in Latin American contexts and in professional squads in Chile [47,48]. Most studies report neuromuscular variables independently or through specific between-group comparisons, without examining the latent organisation of mechanical performance [49,50]. The identification of interpretable neuromuscular components could improve the classification of individual profiles, reduce redundancy between biomechanical variables, and optimise performance monitoring in professional female soccer [51,52]. Therefore, the aim of this study was to characterise the multivariate neuromuscular profile derived from CMJ and IMTP testing and to examine neuromuscular components according to tactical positions in professional female soccer players.

## 2. Materials and Methods

### 2.1. Study Design

To address the study aim, an exploratory study was designed in professional female soccer players classified as tiers 3–4 [53]. The neuromuscular profile was assessed in a single session using countermovement jump (CMJ) and isometric mid-thigh pull (IMTP) tests, recorded with bilateral force platforms. Principal component analysis was used as a strategy to reduce the dimensionality of the mechanical variable set and to extract latent neuromuscular components.

The selected components were interpreted according to their main loadings and synthesised as mechanical dimensions associated with propulsive force, rate of force development, impulse, isometric strength, inter-limb imbalance, and landing control. Individual scores for each component were compared exploratorily according to tactical positions.

### 2.2. Participants

Twenty-three professional female football players aged 19.5 ± 3.8 years, with a body mass of 61.9 ± 6.5 kg, participated in the study. The positional distribution included midfielders, full-backs, centre-backs, forwards, wingers, and goalkeepers from the Chilean professional league. The participants represented all available and eligible players from the professional squad during the assessment period. Accordingly, the sample size reflected a real sporting unit rather than a partial selection of the team. All players trained five to six times per week. They had sustained no lower-limb musculoskeletal injuries during the 90 days preceding data collection and completed the neuromuscular assessment protocol. The participants were informed about the aims, procedures, and requirements of the assessments. All participants provided written informed consent in accordance with the 2013 Declaration of Helsinki [54]. The study was approved by the Ethics Committee of Universidad Adventista de Chile (code 2025-26). Table 1 shows the descriptive characteristics and main neuromuscular variables.

### 2.3. Procedures

**Figure 1:**
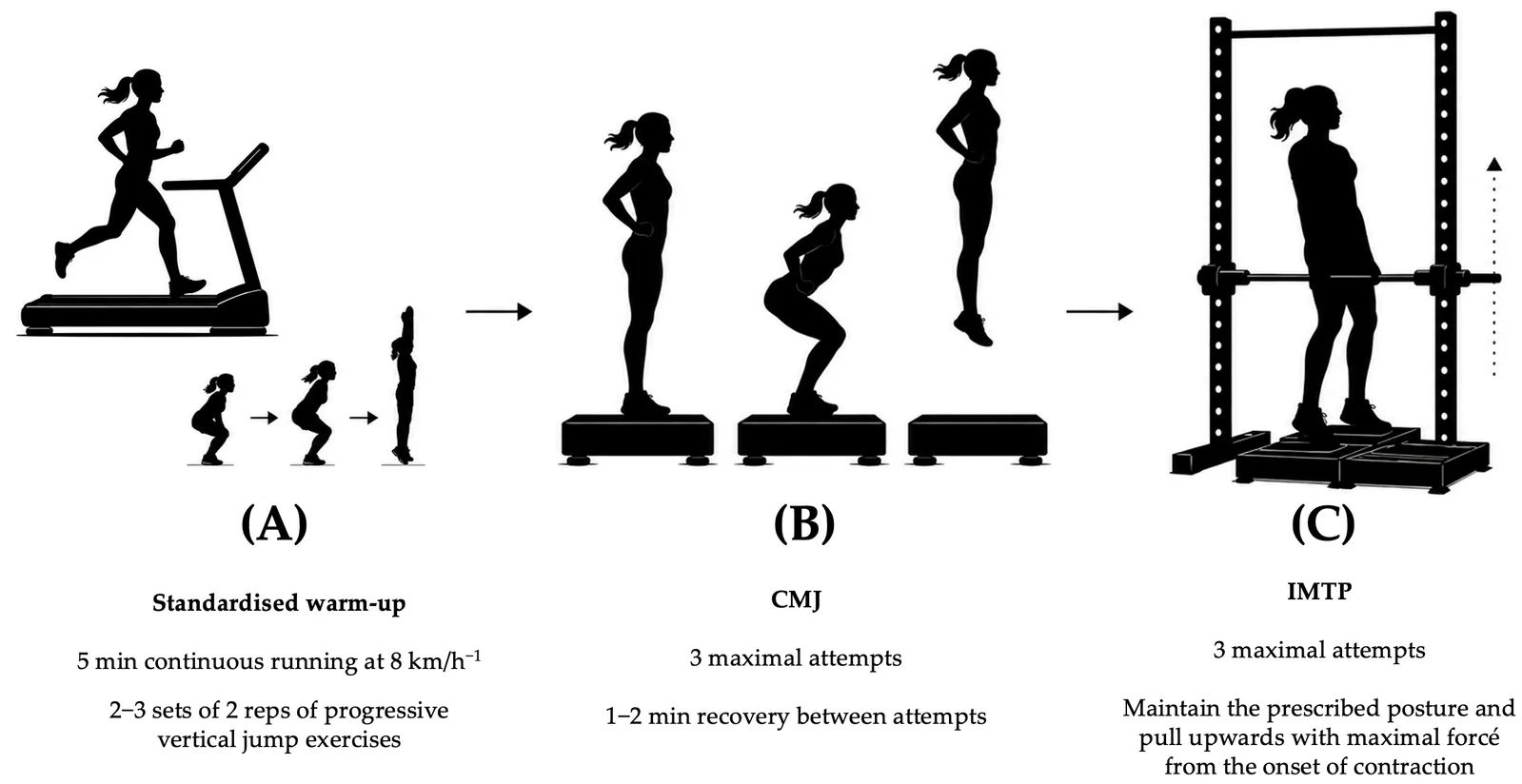
Schematic representation of the neuromuscular assessment procedure.

The assessments were conducted in a single session at an ambient temperature of 21–22 °C. The warm-up comprised 5 min of treadmill running at 8 km·h^−1^, activation exercises, and dynamic mobility exercises for the hips and lower limbs, followed by 2–3 sets of two progressive vertical jumps. The players then completed 3–4 familiarisation trials of the CMJ and IMTP on bilateral Valkyria Trainer Balance force platforms (iVolution, Buenos Aires, Argentina; 1000 Hz), under standardised instructions and supervision by the assessment team (Figure 1).

For the CMJ, three maximal trials were performed with 2 min of recovery between trials. Trials involving technical errors, loss of stability, or incomplete execution were discarded, and valid trials were averaged to reduce intra-individual variability, as reported in previous studies [6,8,19,22,35,55]. For the IMTP, three maximal trials were performed in a position similar to the second pull of the clean, with knee flexion angles of 125–145° and hip flexion angles of 140–150°. Participants were instructed to apply force as rapidly and forcefully as possible, and the trial with the highest peak force was selected to represent maximal voluntary force capacity. The trial-selection criteria were established a priori. Valid CMJ trials were averaged to reduce intra-individual variability and represent typical performance, whereas the IMTP trial with the highest peak force was selected to represent maximal voluntary force-production capacity.

Data were recorded using two portable force platforms (Valkyria Trainer Balance^®^ [VTB^®^]), developed by IVOLUTION^®^ (Sunchales, Argentina). The VTB^®^ system consists of two independent platforms for evaluating the right and left limbs separately. Each platform incorporates a beam-type load cell to capture the unidirectional resultant force along the vertical axis. The signals from these platforms are processed by an analog-to-digital converter and an internal electronic processor with a sampling frequency of 1000 Hz, controlled by the ValkyriaTrainer^®^ software (version 1.1.9, Sunchales, Argentina). The validity and reliability of the system have been previously documented [56]. The platforms were placed on a rigid, level surface, zeroed, and the absence of residual load was verified. Body mass was estimated during the static standing phase prior to movement. During the countermovement jump (CMJ), the takeoff, release, braking, propulsion, and landing phases are automatically identified based on the vertical force relative to body weight. The center of mass velocity was obtained by integrating the net force over time, jump height was calculated from the takeoff velocity, impulses were determined from the area under the force-time curve, and the rate of force development was calculated as the change in force divided by the change in time.

For the IMTP, the onset of contraction was identified when the force exceeded the baseline pretension level. Peak force, mean force, bilateral force, and force at 50, 100, 150, 200, and 250 ms were calculated. The rate of force development was calculated at the 50, 100, 150, and 250 ms intervals.

### 2.4. Variables

The variables derived from the CMJ were organised according to the phases of the movement. For the unweighting phase, temporal and mechanical variables related to the onset of the downward movement were considered. For the braking phase, peak braking force, braking impulse, and braking time were included. For the propulsive phase, peak propulsive force, propulsive impulse, propulsive time, mean force, and variables associated with concentric force production were included. Flight outputs included flight time, jump height, take-off velocity, and the modified reactive strength index. The landing phase included peak landing force, landing stiffness, time to stabilisation, and metrics related to post-contact control.

The variables derived from the IMTP included peak force or maximal isometric force (N), mean force (N), force recorded at specific time windows of 50, 100, 150, 200, and 250 ms (N), and rate of force development (N·s^−1^), calculated both as an absolute value and across early contraction intervals, including 50, 100, and 150 ms. These variables allowed the magnitude of isometric force and the capacity for rapid force production to be characterised under a standardised posture.

As composite indices, inter-limb imbalance indicators were calculated from the percentage difference between limbs using the following formula: [(higher value − lower value)/higher value] × 100. Global imbalance (%), propulsive imbalance (%), and braking imbalance (%) indicators were considered. These indicators were interpreted as complementary measures of the neuromuscular profile, given their possible intra-individual variability and sensitivity to the assessment context.

### 2.5. Statistical Analysis

Descriptive data were expressed as means, standard deviations, medians, and interquartile ranges. Normality was assessed using the Shapiro–Wilk test. PCA was applied to 54 biomechanical variables after preprocessing. All variables were standardised using *z*-scores before analysis. Matrix suitability for PCA was examined using the Kaiser–Meyer–Olkin measure and Bartlett’s test of sphericity. The first five components were retained based on the combined consideration of visual inspection of the scree plot, individual and cumulative explained variance, and the biomechanical coherence of the variables with the largest loadings.

Individual scores for each component were compared according to tactical positions using one-way ANOVA. For each comparison, the *F* statistic, degrees of freedom, exact *p*-value, and eta-squared effect size (η^2^) were calculated. Statistical significance was set at *p* < 0.05. Values of *p* greater than 0.05, including *p* = 0.06, were interpreted as not statistically significant. Analyses were performed using RStudio, version 2024.12.1+563 (Posit Software, Boston, MA, USA).

### 2.6. Data Quality Control

The database was inspected before statistical analysis by two blinded researchers; in cases of disagreement, verification was obtained from a third researcher. The presence of missing values, duplicate records, constant variables, and inconsistencies in player identification was verified. Variables with a standard deviation equal to zero were excluded. Incomplete records for the main variables were removed from the analysis. The tactical position of each player was reviewed and coded before between-group comparisons.

## 3. Results

### 3.1. Data Adequacy and Variance Explained by the Principal Components

The suitability of the correlation matrix for PCA was assessed using the Kaiser–Meyer–Olkin measure and Bartlett’s test of sphericity. The KMO measure indicated insufficient overall matrix adequacy (KMO = 0.479), whereas Bartlett’s test of sphericity was statistically significant (χ^2^(1431) = 3779.87; *p* < 0.001). Given the small sample size relative to the number of variables, these findings were interpreted cautiously, and the PCA was considered exploratory. The solution obtained was considered preliminary and requires validation in a larger independent cohort.

PCA was applied to 54 biomechanical variables after preprocessing. The first five components collectively explained 79.19% of the total variance. PC1 explained 32.05%, PC2 20.73%, PC3 12.85%, PC4 7.25%, and PC5 6.31%. The cumulative variance reached 32.05%, 52.78%, 65.62%, 72.88%, and 79.19%, respectively.

Table 2 presents the individual and cumulative explained variance of the first five principal components.

**Figure 2:**
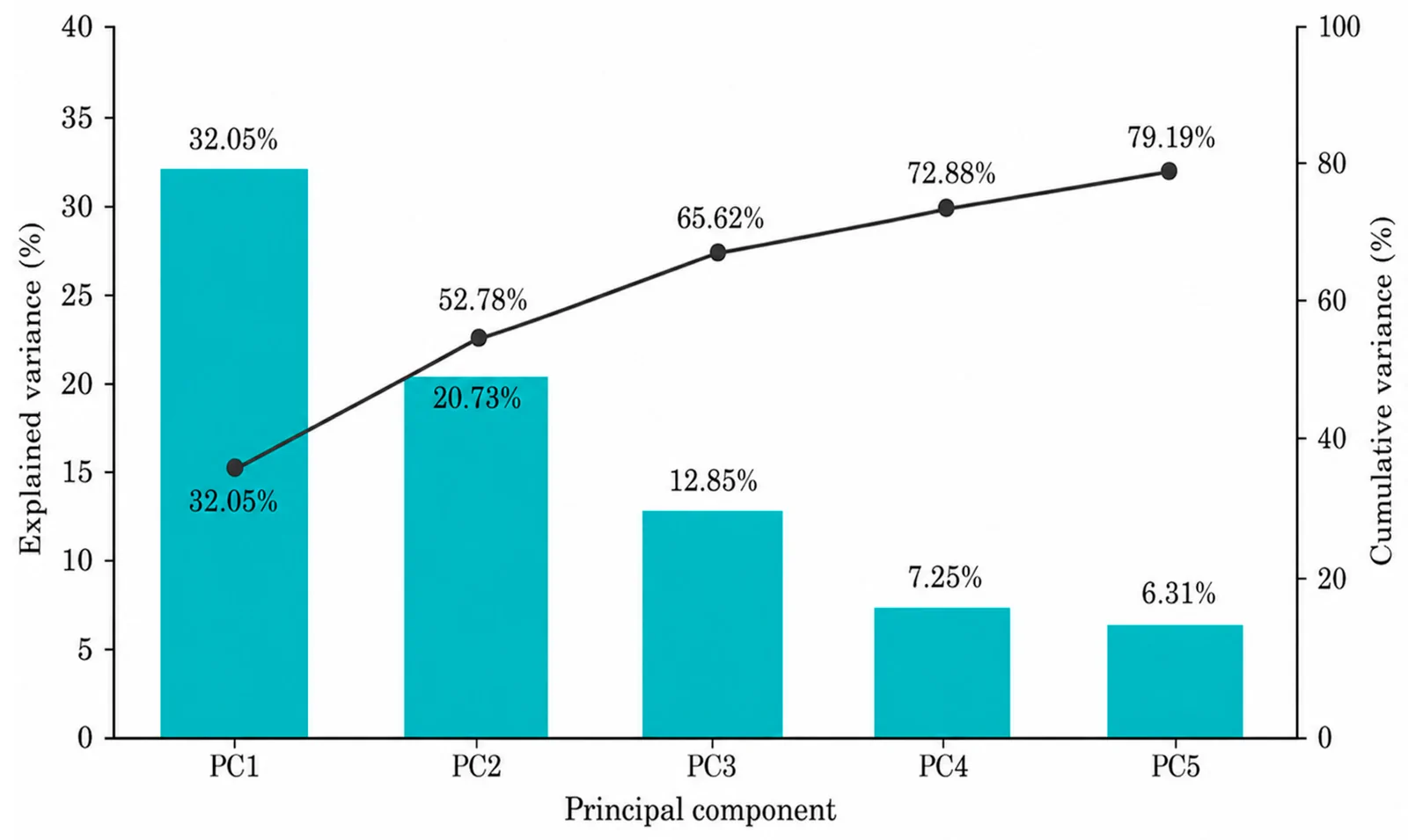
Scree plot of the variance explained by the principal components.

Figure 2 shows the variance explained by each principal component and the cumulative variance of the first five components.

### 3.2. Main Loadings and Component Interpretation

PC1 showed the highest loadings for propulsive RFD, peak propulsive force, left peak propulsive force, right peak propulsive force, and the reactive strength index. It also showed relevant loadings for concentric time, CMJ propulsive phase time, and braking time. PC2 showed the highest loadings for propulsive impulse, positive impulse, mean force, body mass, peak force, force at 250 ms, braking impulse, force at 200 ms, eccentric time, and force at 150 ms. PC3 showed the highest positive loadings for RFD at 100 ms, RFD at 50 ms, RFD at 150 ms, force at 100 ms, and force at 150 ms. The highest negative loadings corresponded to flight time, peak braking force, left peak braking force, right peak braking force, and propulsive impulse. PC4 showed the highest positive loadings for braking imbalance, propulsive imbalance, global imbalance, left peak force, RFD at 250 ms, and RFD at 150 ms. The highest negative loadings corresponded to peak landing force, left peak landing force, right peak force, and body mass. PC5 showed the highest positive loadings for flight time, jump height, peak take-off velocity, time to stabilisation, and unweighting time. The highest negative loadings corresponded to landing stiffness, right peak landing force, global imbalance, left peak force, and peak landing force.

Table 3 summarises the variables with the greatest contribution to each principal component and their biomechanical classification.

**Figure 3:**
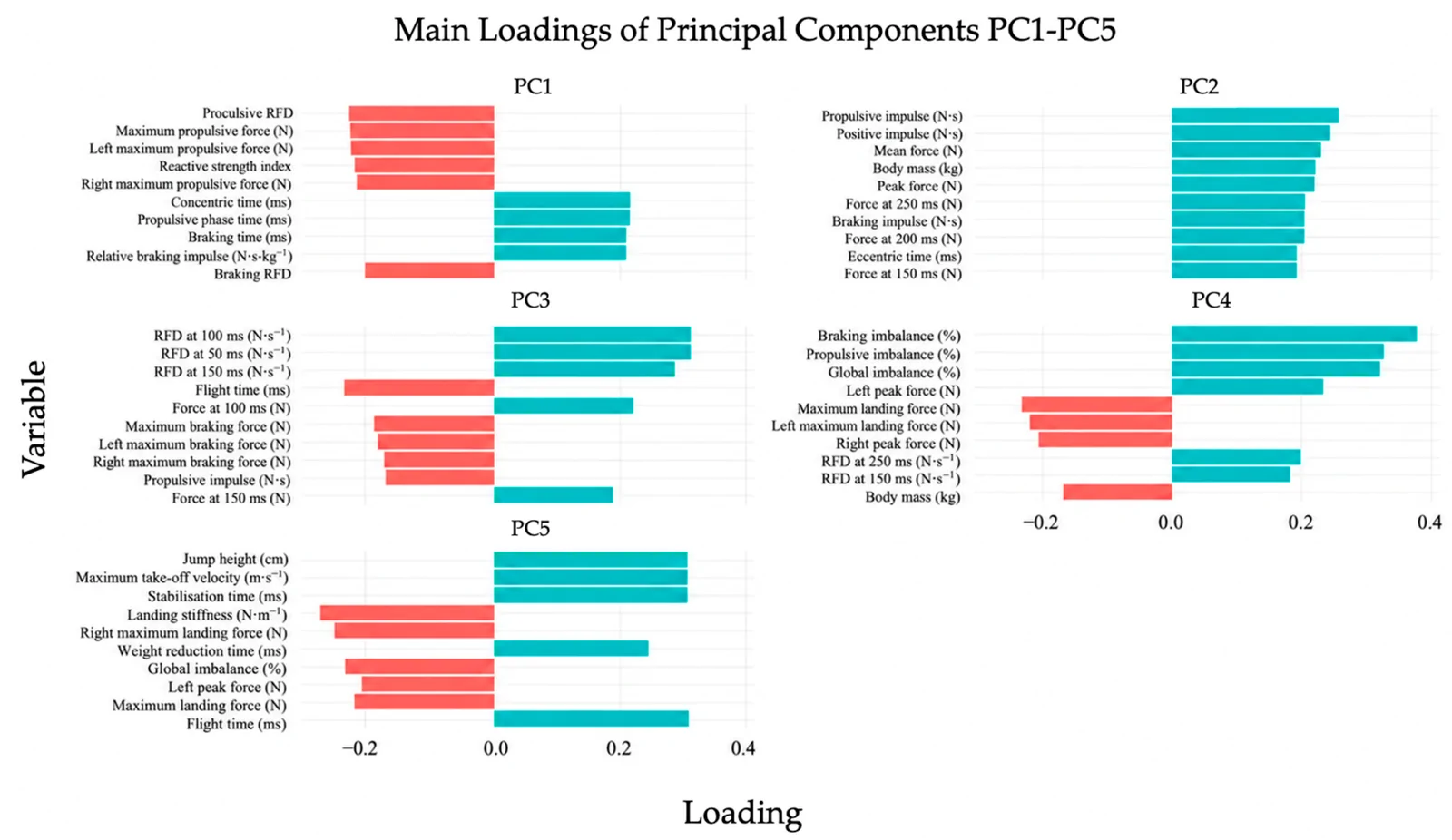
Main loadings of the first five principal components.

The graphical representation of the loadings allowed the relative contribution and direction of the variables within each principal component to be visualised more clearly. Positive and negative loadings reflected the way in which the variables were grouped within each latent dimension, thereby facilitating the biomechanical interpretation of the components. In this regard, PC1 was characterised mainly by propulsive and RFD variables, PC2 by indicators of total force and mechanical impulse, PC3 by early explosiveness variables, PC4 by indicators of bilateral imbalance, and PC5 by variables related to jump performance and landing control. Figure 3 shows the main loadings of the first five PC.

### 3.3. Comparison of Components According to Tactical Positions

The individual scores for the five principal components were compared according to tactical positions using one-way ANOVA. No statistically significant differences were observed for PC1 [F(5,17) = 2.660; *p* = 0.059; η^2^ = 0.439], PC2 [F(5,17) = 1.248; *p* = 0.331; η^2^ = 0.268], PC3 [F(5,17) = 0.634; *p* = 0.677; η^2^ = 0.157], PC4 [F(5,17) = 0.589; *p* = 0.709; η^2^ = 0.148], or PC5 [F(5,17) = 0.528; *p* = 0.752; η^2^ = 0.134]. The *p*-value for PC1 exceeded the predefined significance threshold and was therefore interpreted as not statistically significant. The PC1 result (*p* = 0.059) was interpreted exclusively as non-significant and only as a descriptive tendency across positions. Table 4 shows the comparison of principal component scores according to tactical positions.

**Table 4:** Comparison of principal component scores according to tactical positions.

Principal Component	Interpretation	F Statistic	*p*-Value	η^2^	Statistical Outcome
PC1	Propulsive force and rate of force development	F(5,17) = 2.660	0.059	0.439	Not significant
PC2	Total force and mechanical impulse	F(5,17) = 1.248	0.331	0.268	Not significant
PC3	Early explosiveness	F(5,17) = 0.634	0.677	0.157	Not significant
PC4	Inter-limb imbalance	F(5,17) = 0.589	0.709	0.148	Not significant
PC5	Jump performance and landing control	F(5,17) = 0.528	0.752	0.134	Not significant

Abbreviations: PC, principal component; η^2^, eta squared.

**Figure 4:**
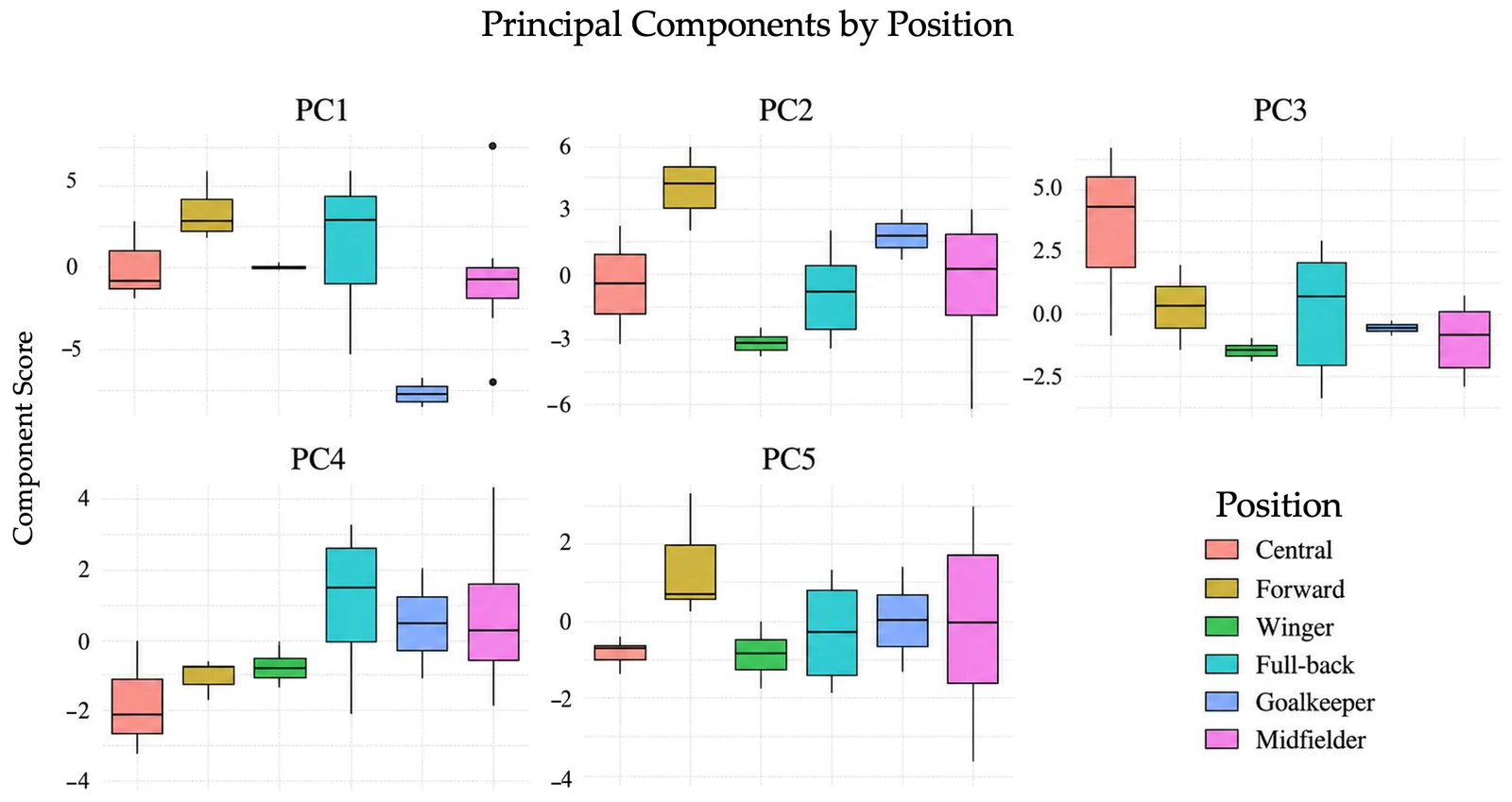
Distribution of principal component scores according to tactical positions.

The graphical distribution of the scores is presented solely for descriptive purposes. Visual separation between tactical positions was not interpreted as evidence of differences because none of the comparisons reached statistical significance. Figure 4 shows the distribution of the individual scores for each component according to tactical position.

## 4. Discussion

The novelty, relevance, and significance of this study lie in the integration of variables derived from the CMJ and IMTP within a single multivariate framework, allowing complex neuromuscular information to be synthesised into interpretable dimensions for professional female football players. This approach provides a broader representation of neuromuscular performance than the isolated analysis of mechanical variables and addresses the need to consider force production [55,57], the speed of force application [55,58], the organisation of impulse and propulsive phases [58,59], eccentric control [5,37], and bilateral force distribution [60,61] collectively. The aim of the study was to characterise the multivariate neuromuscular profile derived from CMJ and IMTP assessments and to examine neuromuscular components according to tactical positions. The first five components explained 79.19% of the total variance and represented rapid propulsive capacity, force and impulse magnitude, early explosiveness, inter-limb imbalance, and jump and landing performance. No statistically significant differences were observed between tactical positions for any of the components analysed. Consequently, the identified components were interpreted as an exploratory rather than confirmatory solution, whose stability should be validated in a larger independent cohort.

The organisation of the first principal component around propulsive force and rate-of-force-development variables identified rapid propulsive capacity as a relevant dimension of the neuromuscular profile. This interpretation is consistent with research highlighting the value of force-time variables for characterising physical performance in football players [5,14,57]. Accelerations, decelerations, changes in direction, jumps, and physical duels require high force production within short time intervals [62,63]. Jump height or maximal force analysed independently may provide an incomplete representation of neuromuscular function because they do not fully describe the temporal organisation of force application [5,55,58]. The combined assessment of propulsive force and rate of force development may therefore be more sensitive for identifying inter-individual mechanical differences, neuromuscular fatigue, and training adaptations than global jump variables [9,55,64].

The mechanical dimension represented by PC2 complements the explosive characteristics of PC1 by reflecting the magnitude and duration of force application [55,58]. Impulse integrates the force produced and the duration of its application during a specific phase of movement, providing information that cannot be obtained exclusively from maximal force or jump height [58,59]. In football players, this variable may contribute to characterising the ability to displace the centre of mass and sustain force production during explosive actions [5,14,57]. The distinction between rapid force application and total force-impulse magnitude indicates that athletes may achieve similar outcomes through different neuromuscular strategies [38,55,58]. This interpretation supports recommendations to avoid exclusively univariate analyses, particularly when athletes with comparable CMJ performance may present heterogeneous underlying mechanical profiles [9,38,55].

The dimensions associated with early explosiveness, inter-limb imbalance, and jump and landing control provide complementary information on specific neuromuscular qualities. Early force production is particularly relevant during the initial steps of acceleration, physical contact, changes in direction, and rapid defensive responses, in which the time available for force application is limited [11,15,57]. Inter-limb imbalance indicators may contribute to individual monitoring by identifying changes in force distribution between limbs [60,61]. These measures should not be interpreted as isolated diagnostic thresholds because percentage differences may vary according to the task, testing session, calculation method, and individual athlete characteristics [58,60,61]. The combined interpretation of flight time, take-off velocity, time to stabilisation, stiffness, and landing forces allows a more comprehensive assessment of vertical-jump performance by considering both propulsive production and post-contact force absorption and control [5,8,36].

No statistically significant differences were identified between tactical positions. The graphical separation observed between some positional groups, including forwards, should be considered descriptive and should not be interpreted as evidence of a positional effect. Previous studies have reported more consistent positional differences in external load, distance covered, accelerations, decelerations, and locomotor demands than in strength, jump, or neuromuscular-performance variables [62,63,65]. The absence of differences may be related to the competitive homogeneity of the squad, the small number of players within each tactical category, and their shared exposure to similar physical-conditioning programmes. Positional comparisons require larger samples, consistent tactical classifications, and control for competitive level, age, sporting experience, maturational status, and previous training load [53,62,66]. The multivariate approach used may therefore be more appropriate for individual profiling than for establishing position-specific reference values. The integration of CMJ, IMTP, and PCA may help organise multiple force-platform variables, identify individual mechanical characteristics, and support decision-making in the physical preparation of professional female football players [38,55,57]. The PC1 *p*-value of 0.059 was interpreted as non-significant and only as a statistical trend.

From an applied perspective, the identified components may inform training prescription by distinguishing athletes who require greater emphasis on rapid force production, total force and impulse development, early explosiveness, bilateral force distribution, or landing control. Repeated assessments may also help determine whether changes associated with training, fatigue, injury, or return-to-play processes occur within specific neuromuscular dimensions rather than being reflected exclusively by changes in jump height or maximal force. A strength of the study was the combined assessment of dynamic and isometric force production using bilateral force platforms and a multivariate strategy designed to reduce redundancy among correlated variables.

The principal limitation was the sample size of 23 athletes relative to the 54 variables included in the PCA. Although the sample comprised the entire available professional squad, this low participant-to-variable ratio may produce components and loadings that are sensitive to the specific characteristics of the sample. The KMO measure indicated insufficient matrix adequacy, while interpretation of Bartlett’s test was limited by the singularity of the correlation matrix. Additional limitations include the small number of players within each tactical position, the inclusion of a single squad, the cross-sectional design, and the absence of external validation or resampling of the solution. The components should therefore be interpreted as preliminary descriptive hypotheses rather than as a confirmatory or generalisable neuromuscular structure. The selection of five components based on inspection of the scree plot and the biomechanical interpretation of the loadings involved a degree of subjectivity. The absence of parallel analysis limits the statistical support for the number of components retained; therefore, this approach should be incorporated into future research.

Future studies should incorporate multicentre designs, larger samples, longitudinal assessments, bootstrap resampling or cross-validation procedures, and replication of the PCA at different stages of the competitive season. Integrating external and internal load variables, injury history, competitive experience, and sport-specific performance outcomes would allow the stability, clinical relevance, and practical utility of the identified components to be evaluated. Parallel analysis and external validation in a larger independent cohort should be included to confirm the number, stability, and interpretability of the components.

## 5. Conclusions

Principal component analysis enabled 54 variables derived from the CMJ and IMTP to be synthesised into five biomechanically interpretable dimensions, which collectively explained 79.19% of the total variance. These dimensions were associated with rapid propulsive capacity, force and impulse magnitude, early explosiveness, inter-limb imbalance, and jump and landing performance. No statistically significant differences were identified between tactical positions. The integration of CMJ, IMTP, and PCA may facilitate the organisation of multiple force-platform variables and contribute to the individual monitoring of professional female football players. Given the sample size, the high dimensionality of the dataset, and the insufficient adequacy indicated by the KMO measure, the findings should be considered preliminary until validated in larger, multicentre samples. Therefore, the findings should be regarded as strictly exploratory rather than confirmatory until they have been validated in a larger independent cohort.

## Figures and Tables

**Table 1:** Descriptive characteristics and main neuromuscular variables of the players.

Variable	Mean ± SD	Median	IQR
Age (years)	19.5 ± 3.8	19.0	17.0–20.0
Body mass (kg)	61.9 ± 6.5	62.0	58.5–67.0
CMJ height (cm)	30.4 ± 2.7	31.0	29.3–31.9
Take-off velocity (m·s^−1^)	2.44 ± 0.11	2.47	2.40–2.50
Modified RSI (a.u.)	0.39 ± 0.08	0.38	0.34–0.42
Maximal propulsive force (N)	1494.0 ± 246.9	1521.7	1292.7–1652.7
Propulsive RFD (N·s^−1^)	5419.6 ± 1556.9	5283.0	4384.0–6312.3
Maximal propulsive power (W)	2987.1 ± 514.0	3083.7	2653.5–3342.2
Landing peak force (N)	2680.1 ± 622.1	2655.0	2295.0–3138.0
Time to stabilisation (ms)	1060.8 ± 366.8	983.3	828.5–1126.7
IMTP peak force (N)	1985.9 ± 225.1	2005.0	1789.0–2098.5
IMTP relative force (N·kg^−1^)	17.6 ± 3.1	18.5	15.1–19.5
Force at 100 ms (N)	1254.2 ± 229.6	1252.5	1115.8–1385.0
Force at 250 ms (N)	1516.5 ± 216.5	1486.5	1397.2–1645.2
Global inter-limb imbalance (%)	14.8 ± 8.7	11.4	8.9–20.2
Propulsive inter-limb imbalance (%)	5.5 ± 4.8	3.5	1.6–9.9
Braking inter-limb imbalance (%)	9.7 ± 7.5	9.5	4.1–13.6

Abbreviations: CMJ: countermovement jump; IMTP: isometric mid-thigh pull; RFD: rate of force development; RSI: reactive strength index; SD: standard deviation; IQR: interquartile range; a.u.: arbitrary units.

**Table 2:** Explained variance of the first five principal components.

Principal Component	Explained Variance (%)	Cumulative Variance (%)	General Interpretation
PC1	32.05	32.05	Propulsive force and rate of force development
PC2	20.73	52.78	Total force and mechanical impulse
PC3	12.85	65.62	Early explosiveness
PC4	7.25	72.88	Bilateral asymmetries
PC5	6.31	79.19	Jump performance and landing control

Abbreviations: PC: principal component.

**Table 3:** Main variables and the biomechanical interpretation of the principal components.

Principal Component	Variables with the Greatest Contribution	Biomechanical Interpretation
PC1	Propulsive RFD, maximum propulsive force, left/right maximum propulsive force, reactive strength index, execution times	Explosive force and propulsive capacity
PC2	Propulsive impulse, positive impulse, mean force, peak force, body mass, force at 50–250 ms	Total force and mechanical impulse
PC3	RFD at 50 ms, RFD at 100 ms, RFD at 150 ms, early force variables	Early explosiveness
PC4	Braking muscle imbalance, propulsive muscle imbalance, global muscle imbalance, left/right peak force	Bilateral asymmetries
PC5	Flight time, jump height, take-off velocity, stabilisation time, landing stiffness	Jump performance and landing control

Abbreviations: PC: principal component; RFD: rate of force development.

## Data Availability

The raw data supporting the conclusions of this article will be made available by the authors upon request.
